# Supplementary material for: Content-rich biological network constructed by mining PubMed abstracts
Source: BMC Bioinformatics. 2004 Oct 8;5:147. doi: 10.1186/1471-2105-5-147 (PMC528731; doi:10.1186/1471-2105-5-147)
Supplement: Additional File 2 — The original results of the above study (non-essential files are deleted to keep the file size under the limit set by BMC bioinformatics). [file 1471-2105-5-147-S2.bz2 › chilibotAdditionalFile2/dip10/215ID7790364E721/html/DTR_CD9.html]

 


 **DTR** and **CD9** 
  
Found 31 abstracts in PubMed, retrieved 10.  
 

 What does Google say? 
 PDF only 
| .edu only 

---

**Interactive relationship** (e.g. stimulation, inhibition, etc)

**Neutral relationship**- These three amino acid residues were deduced to be located on the head domain of the second extracellular loop, suggesting that interactions of  **CD9**  with  **DTR**  or DT at the domain containing these three amino acids were important for the upregulation of DT binding.  Ref: 11735113 Biochem Biophys Res Commun, 2001
- Human and monkey  **CD9**  upregulates DT binding activity of  **DTR** , while mouse  **CD9**  has no upregulation activity.  Ref: 11735113 Biochem Biophys Res Commun, 2001
- They also overexpressed  **CD9** , a tetraspanin that binds to the heparin binding domain of HB EGF  [ **DTR** ]  and is critical for promoting ErbB1 activation by HB EGF  [ **DTR** ] .  Ref: 11971193 Oncogene, 2002
- **CD9**  associates with a diphtheria toxin receptor  [ **DTR** ]   **DTR**  that is identical to the membrane anchored form of heparin binding EGF like growth factor  [ **DTR** ] .  Ref: 11735113 Biochem Biophys Res Commun, 2001
- The tetra membrane spanning protein  **CD9**  forms a complex with a membrane anchored heparin binding epidermal growth factor like growth factor  [ **DTR** ]  HB EGF  [ **DTR** ]  and integrin alpha3beta1 in some human and monkey cell lines.  Ref: 11259446 J Histochem Cytochem, 2001
- **CD9**  associates with diphtheria toxin receptor  [ **DTR** ]   **DTR** , which is identical to the membrane anchored form of heparin binding EGF like growth factor  [ **DTR** ]  proHB EGF.  Ref: 11235900 Cell Struct Funct, 2000
- Human and monkey  **CD9**  upregulate the diphtheria toxin DT binding activity of human heparin binding EGF like growth factor  [ **DTR** ] , while chick  **CD9**  lacks the ability to upregulate the DT binding activity.  Ref: 10948653 Kurume Med J, 2000

**Non-interactive relationship** (e.g. studied together, co-existance, homology, etc.)

- These data indicate that in the XG 1 and XG 14 IL 6 dependent myeloma cell lines, the  **CD9**  HB EGF  [ **DTR** ]  erbB1 and the IL 6 IL 6R gp130 pathways cooperate synergistically to trigger myeloma cell growth.  Ref: 11971193 Oncogene, 2002
- We show here the immunohistochemical distribution of  **CD9** , HB EGF  [ **DTR** ] , and integrin alpha3beta1 in normal human tissues.  Ref: 11259446 J Histochem Cytochem, 2001
- **CD9**  upregulates the diphtheria toxin DT binding activity of  **DTR**  proHB EGF, while CD63 does not upregulate the DT binding activity in spite of the fact that this protein also associates with  **DTR**  proHB EGF on the cell surface.  Ref: 11235900 Cell Struct Funct, 2000
- A function blocking antibody against  **CD9**  DRAP27  [ **CD9** ]  MRP 1  [ **CD9** ] , a cell surface binding partner of the membrane form of HB EGF  [ **DTR** ] , also stimulated human prostate stromal cell proliferation.  Ref: 11125426 J Urol, 2001
- RESULTS HB EGF  [ **DTR** ] , its receptors HER 1 and HER 4, and the associated proteins  **CD9** , CD44, and integrin alpha 3 beta 1 were expressed by HPMCs and peritoneal macrophages.  Ref: 11168942 Kidney Int, 2001
- The XG 6 and XG 13 myeloma cells that were not significantly sensitive to HB EGF  [ **DTR** ]  antagonists, poorly expressed HB EGF  [ **DTR** ] , ErbB1 and  **CD9**  genes or proteins.  Ref: 11971193 Oncogene, 2002
- Significance of the association between heparin binding epidermal growth factor like growth factor  [ **DTR** ]  and  **CD9**  in human gastric cancer.  Ref: 11920609 Int J Cancer, 2002
- Juxtacrine activity of proHB EGF the membrane anchored form of HB EGF  [ **DTR** ]  has been shown to be significantly potentiated when it is coexpressed with  **CD9**  in vitro.  Ref: 11920609 Int J Cancer, 2002
- HB EGF  [ **DTR** ]  gene expression and protein production in human gastric cancers was investigated, and EGF receptor and  **CD9**  expressions were also evaluated.  Ref: 11920609 Int J Cancer, 2002
- Distribution of  **CD9** , HB EGF  [ **DTR** ] , and integrin alpha3beta1 was similar in various tissues, including transitional epithelium, squamous epithelium, thyroid follicular epithelium, adrenal cortex, testis, smooth muscle, and stromal fibrous tissue.  Ref: 11259446 J Histochem Cytochem, 2001
- GPCR activation enhances the association of KUZ and its substrate HB EGF  [ **DTR** ]  with tetraspanin  **CD9** .  Ref: 12119356 J Cell Biol, 2002
- Immunohistochemical distribution of  **CD9** , heparin binding epidermal growth factor like growth factor  [ **DTR** ] , and integrin alpha3beta1 in normal human tissues.  Ref: 11259446 J Histochem Cytochem, 2001
- **CD9**  was distributed in the myelin, but HB EGF  [ **DTR** ]  was found in the axon of the peripheral and central nervous systems.  Ref: 11259446 J Histochem Cytochem, 2001
- In conclusion, there is a possibility of complex formation and functional cooperation of  **CD9**  with HB EGF  [ **DTR** ]  and or integrin alpha3beta1 in several tissues.  Ref: 11259446 J Histochem Cytochem, 2001
